# Program for data extraction in primary health records: a valid tool for knowledge production in general practice?

**DOI:** 10.1186/s13104-020-4887-7

**Published:** 2020-01-10

**Authors:** Martin Holte, Jostein Holmen

**Affiliations:** 1 Risvollan Health Center, Ingeborg Aas’ veg 2, 7036 Trondheim, Norway; 2 HUNT Research Center, Department of Public Health and Nursing, Norwegian University of Science and Technology (NTNU), Forskningsvegen 2, 7600 Levanger, Norway

**Keywords:** Extraction program, Health records, General practice, Research, Quality control

## Abstract

**Objectives:**

Research in general practice demands it-tools which give the practitioner trusty results. Medrave 4 is a program designed for extraction of data from all areas of the health record. We wanted to do research on the database in a health center, but found no proof of the quality of the data extracted by Medrave 4. Today the database contains about 40,000 records. In this study we wanted to examine if the program could extract correct data.

**Results:**

From the database 20 records were randomly selected from five different time periods, making a total of 100 records. 14 records did not meet the inclusion criteria, resulting in 86 records included in the study. In phase one these variables were registered manually from the records: Age, gender, systolic and diastolic blood pressure (from free text) and six different laboratory tests. In phase two, Medrave 4 extracted the same variables from the same records. Medrave 4 found correct systolic and diastolic blood pressure values in 79 records (92%). The laboratory results were extracted correct in all 86 records (100%). We conclude that Medrave 4 can be a useful tool in quantifying the work of general practitioners.

## Introduction

There is an increasing interest in knowledge production and quality assurance in general practice [1], but a basic premise is the opportunity to have an overview of own practice. The general practitioners therefore need to quantify the content of their practice, but most record systems do not allow such overview. Several studies are performed to evaluate the data quality of the primary health records [2, 3], but few have evaluated the programs used for extraction of data, and we found no one performed in Scandinavia, but one in UK [4]. Medrave 4 is a program designed for extraction of data from primary health records, and we planned using this program in a study of hypertension treatment in a health center [5]. When asking for documentation of whether the program in fact was able to extract the data we wanted, we understood that no independent validation study of the program had been conducted. The health center we wanted to study has a 40 years history, using different electronic record systems, and we wanted to extract data from the last 25 years. As it is crucial to know to which extent the program can extract the correct data, we wanted to do a validation study of the program: To which extent can Medrave 4 extract the data elements we ask for? Are there limitations regarding how old the data are, or regarding which record system or version of record system where data are registered?

## Main text

### The extraction program

Medrave 4 was launched in 2012, based on development of Rave Data System launched already in 1996 by a general practitioner (GP) [6]. The motivation behind developing the program was the need for an analytic tool in describing general practice. The distributor has described these aims for the program: (1) identification of patients with potential need of case management based on the licensed John Hopkins ACG model, (2) a medical indicator platform enabling GPs to visualize their own performance, and to compare with other GPs anonymously (benchmark), and (3) to give health officers and government the possibility to visualize comparisons of indicators between municipalities without exposing patients, GPs or clinical practices.

Today, Medrave 4 is based on a Microsoft DOT-framework and a SQL server database technology, and is a reporting and a statistics program. The program is adjusted to fit the record systems System X, CGM general, Infodoc Plenarion, WinMed2 and Profdoc Vision [7]. The adjustments are performed in collaboration with the Norwegian Association of General Practice.

Technically, the program is installed in the same data network, typically on the same server, as the medical record system. A SQL server is used, and data are read from the record system database into the Medrave 4 statistical database every night. For presenting the extracted data, a local intranet application is used. This means that all data are located on the server in the health center, and the working stations have no direct connection to the statistical database. The users may have access to the Medrave 4 application with user name and password. The reports are presented as tables and graphs, and it is possible to click to see which patients who are represented in the data (Drilldown). These functions are available for own patients only, but not for patients belonging to other physicians in the health center, provided the user is not a system administrator. All data are labelled with patient identity, date, time of the day, data user and the type of report displayed. The program can also trace and monitor high risk patients.

Medrave claims that the program can extract data from all areas in the patient records, even in the free text, and it is used in previous projects [8, 9].

### The medical records

Risvollan health center in Trondheim, Norway, was established in 1974 with approximately 9500 citizens affiliated to the health center, distributed on six physicians. The physicians have been stable, i.e. the same five physicians have stayed for 38 years, and one physician stayed for 20 years (up to year 2010). The medical records were digitalized already in 1985–1986, and today the health center contains about 40,000 medical records. Naturally, the record systems are updated and changed several times, resulting in mainly four time periods after equally numbers of conversions:

- Period 1: 1986–1996: Norstar (COSTAR—developed in USA, based on MUMPS program language, and adjusted to Norwegian routines at Risvollan health center) [10].
- Period 2: 1992–2005: Profdoc (Hove Medical System) [11].
- Period 3: 2006–2009: Profdoc Vision (Hove Medical System [11].
- Period 4: 2010—up to this day: System X (Hove Medical System) [12].

All physicians in the health center are specialists in general practice, and they have always been engaged in professional updating and standardized procedures in clinical examination and laboratory procedures.

### Blood pressure measurements

The procedures were based on the standardized procedures described by the Norwegian College of General Practice [13]. Validated sphygmomanometers were used. Until year 2005 mercury manometers were used, then there was a change to electronically sphygmomanometers. All physicians used same type of equipment, except one doctor, who continued to use mercury sphygmomanometers.

### Laboratory measurements

The blood samples were drawn according to standardized procedures, but in some cases triglycerides might have been taken in a non-fasting state. From 1974 to 2002 the health center sent the blood samples to The Regional Hospital in Trondheim/St. Olav Hospital, and since 2002 the samples were sent to Fürst Laboratory, Oslo. In a 2–3 years period around year 2000 some analyses were performed in own laboratory using Reflotron and Cholestec.

### Selection of variables

We selected these variables to be extracted from the medical records: Age, gender, systolic blood pressure (SBP), diastolic blood pressure (DBP), kreatinin, total cholesterol, high density lipoprotein (HDL), triglycerides and low density protein (LDL).

### Selection of patients

System X found 100 randomly selected patients from medical records according to these criteria:

- Inclusion criteria: Valid values on the chosen variables (LDL was not analysed until 1995). Inactivated records were also included; i.e. records on patients who had moved or died.
- Exclusion criteria: Patients who declined participation, had wrong identity number or who did not meet the inclusion criteria.

In order to include record from different periods of time, the 100 selected records were distributed with 20 record from each of five periods by 5 years (according to date of consultation): Group 1: 1987–1991, group 2: 1992–1996, group 3: 1997–2001, group 4: 2020–2006, group 5: 2007–2012. Then, the registration of data was performed in two phases:

### Phase 1: Manually registration

One of the authors (MH) gained access to the selected records in System X, searched each record manually and registered values of the selected variables.

### Phase 2: Extraction by Medrave 4

After finishing the manually registration, Medrave 4 extracted the same variables from the same records.

In their marketing, Medrave guarantee correct extracting of data up to 10 years back in time. Because we in this study wanted to find data back to 1987, Medrave 4 needed some adjustments. During this process Medrave Software had only reading access to the System X database.

### Results

Four patients rejected participation. Additionally, two patients were excluded due to lacking blood pressure values in the 5 year time period, and eighth patients had no laboratory values. In the remaining 86 records included in the study, Medrave 4 found SBP and DBP in 79 patients. In the 20 eldest records (from period 1: 1987–1991) Medrave did not find blood pressure values in three of the records. Also in period 4 and 5 (2002–2012) Medrave 4 did not find blood pressure values in three records. Regarding the laboratory measurements, all values registered manually and values extracted by Medrave 4 were identically (Table 1).

**Table 1:** Data from medical records registered manually in System X and data extracted from the same records by Medrave 4

	System X-manually		Medrave 4	
	N	Mean	N	Mean
Age (years)	86	62.2	88	62.2
Female	33		33	
Male	53		53	
SBP (mmHg)	86	144.5	79	146.4
DBP (mmHg)	86	85.8	79	87.0
Creatinine (μmol/L)	86	83.9	86	83.9
Total cholesterol (mmol/L)	86	6.1	86	6.1
HDL (mmol/L)	86	1.36	86	1.36
LDL (mmol/L)	86	2.79	86	2.79
Triglycerides (mmol/L)	86	1.78	86	1.78

Number of records (N) and mean of values for patients with valid values. Medrave 4 did not find SBP and DBP in seven records

In some patients the date of registration of laboratory values were not identical in System X and Medrave 4. In six patients Medrave 4 found the correct values 1 day prior to the day they were registered in System X, in one patient 8 days prior and one patient 21 days prior.

### Discussion

The data elements chosen for this study were selected because we initially wanted to study the hypertension treatment in the health center. We were most excited if Medrave 4 would be able to find the correct blood pressure values, because these usually are written in the free text. Laboratory values are usually registered in the laboratory picture, and should therefore be easier to extract. Standard software for Medrave 4 is supposed to extract data up to 10 years old, but because we wanted to extract data up to 25 years old, the software needed an adjustment.

In the 20 oldest records, Medrave 4 did not find blood pressure values in three records, and the same was the case in the more recent registered records from 2002 to 2012. The laboratory values extracted by Medrave 4 was identically with the values registered manually.

For some laboratory values there was a discrepancy regarding the date of registration. After consulting the software producer we found that this was due to different routines for registering laboratory answers. There are three options for registering dates of laboratory answers: Date of requisition, date of analyses and date when laboratory answers are received. Medrave used date of requisition. This might create confusion if there is time lag between requisition and blood sampling. To reduce the risk of misinterpretation, we therefore recommend Medrave to use date of analyses.

Generally, much emphasis has been on the data quality of the primary health records [2, 3], but we found only one study who evaluated a program used for extraction of data, like we have done in the present study [4]. In our search for other similar extraction programs for comparison, we found that in Sweden, several programs like “Quick View” and “Power BI” were evaluated, but the conclusion was that necessary adjustments of these programs would be very demanding [14]. Therefore, as per today we found no other program available that is comparable with Medrave 4.

According to the distributor, Medrave 4 is now used by more than 750 health centers in Sweden, and in 95 general practices in Norway [6]. In 2018 the Norwegian Directorate of Health initiated a pilot study for quality improvement in general practice, including six municipalities, using Medrave 4 as a tool [15].

### Conclusion

As a conclusion, we found no relevant program for comparison, but our data indicate that Medrave 4 can extract complete and correct laboratory data from primary health records. After some adjustments, Medrave 4 could find laboratory data as long back as 1987 independent of software version. Blood pressure values that were registered in free text, were correctly extracted from 92% of the records. With a possible reservation that the program might not find all data in the free text, our data indicate that Medrave 4 can be a useful tool in getting an overview and quantifying the work of general practitioners.

## Limitations

- The study was performed in one health center in Norway.
- Only SBP and DBP was extracted from the free text section.
- A limited number of laboratory tests was extracted.

## Data Availability

The extracted raw data are available on request.

## Abbreviations

GP: general practitioner
SBP: systolic blood pressure
DBP: diastolic blood pressure
HDL: high density lipoprotein
LDL: low density protein

## References

[1] Regulation of management and quality improvement in health, chap 2, §7. Norway: Ministry of Health and Care Services. 2016. https://lovdata.no/dokument/LTI/forskrift/2016-10-28-1250. Accessed 7 Jan 2020.

[2] Hansell A (1999). Use of the General Practice Research Database (GPRD) for respiratory epidemiology: a comparison with the 4th Morbidity Survey in General Practice (MSGP4). Thorax.

[3] Horsfield P, Teasdale S (2003). Generating information from electronic patient records in general practice: a description of clinical care and gender inequalities in coronary heart disease using data from over two million patient records. Inform Prim Care.

[4] Hammersley Victoria, Meal Andrew, Wright Lynne, Pringle Mike (1998). Using MIQUEST in General Practice. Journal of Innovation in Health Informatics.

[5] Medrave Software AB. Background. Medrave Software. 2017. https://www.medrave.com/about. Accessed 7 Jan 2020.

[6] Medrave Software AB. A cornerstone in quality improvement. Medrave Software. 2017. https://www.medrave.com/. Accessed 7 Jan 2020.

[7] Medrave Softwarre AB. What is Medrave 4. Medrave Software. 2017. https://www.medrave.com/product. Accessed 7 Jan 2020.

[8] Espelid I, K Simonsen, Skaare AB, Willumsen T, Straand J, Gjelstad S, Klock KS, Berggreen E, Rørtveit G, Willumsen T (2018). General practitioners and dentists—a pilot project based on data extraction from electronic patient records. Nor Tannlegeforen Tid.

[9] Skaare AB, K Simonsen, Espelid I, Straand J, Gjelstad S, Klock KS, Berggreen E, Rørtveit G, Willumsen T (2018). Medication-induced dry mouth in two selected patient groups: A pilot study among general practitioners and dentists based on data collected from electronic patient records. Nor Tannlegeforen Tid.

[10] UHSU Clinfowiki. Computer stored amulatory record. COSTAR. 2015. http://clinfowiki.org/wiki/index.php/Computer_Stored_Ambulatory_Record. Accessed 7 Jan 2020.

[11] Langballe F. 20 år med EDB-journal - 14 år med Profdoc Utposten, 1999. p. 9–11.

[12] System-X. Hove Medical. https://hovemedical.no/system-x/.

[13] Høyt blodtrykk. NSAMs handlingsprogram, in Utredningsrapport nr U 3-1993. In: Holmen J, editor. Statens institutt for folkehelse: Verdal; 1993.

[14] anders.o.olsson@regionvarmland.se. Personal communication. 2019.

[15] Norwegian Directorate of Health. Pilot for structured multidisciplinary follow-up team. 2018. https://helsedirektoratet.no/pilot-for-strukturert-tverrfaglig-oppfolgingsteam. Accessed 7 Jan 2020.

